# A Rare Case of Rib Osteoblastoma: Imaging Features and Review of Literature

**DOI:** 10.5812/iranjradiol.7108

**Published:** 2013-08-30

**Authors:** Shokouh Taghipour Zahir, Naser Sefidrokh Sharahjin, Saeed Kargar

**Affiliations:** 1 Department of Pathology,Shahid Sadoughi University of Medical Sciences, Yazd, Iran; 2 Department of Radiology, Shahid Sadoughi University of Medical Sciences, Yazd, Iran; 3 Department of Surgery, Shahid Sadoughi University of Medical Sciences, Yazd, Iran

**Keywords:** Ribs, Osteoblastoma, Bone Neoplasms, Osteoma, Osteoid

## Abstract

Osteoblastoma is a rare benign, but locally aggressive bone tumor with rare malignant transformation. It mostly affects the vertebral column and long bones. Radiographically, it is seen as an expansile, oval, sclerotic or lytic mass-like lesion with well-defined borders, although sometimes it may mimic a malignant tumor such as osteogenic sarcoma by its irregular borders. Herein, we report a case of osteoblastoma in a 22 year-old man with a long history of back and neck pain accompanied with neck stiffness. On the routine chest X-ray, the salient lesion appeared as an expansile, oval, sclerotic mass with well-defined borders and speckled calcification without any internal lucency and periosteal reaction, involving the posterolateral aspect of the first left thoracic rib, a rare anatomical site. Despite the unusual location, osteoblastoma should be considered in the differential diagnosis of a solitary rib lesion.

## 1. Introduction

Osteoblastoma is a rare benign bone tumor that affects males more than females with a ratio of 2.5-1. Its peak incidence is in the second decade, and it is rare before 10 years and after 30 years of age (1). For many years osteoblastoma was named as giant osteoid osteoma denoting its histopathological similarity with osteoid osteoma but it reveals a larger nidus (2, 3). Lesions with a nidus size more than 2 cm are considered as osteoblastoma (1-4). It accounts for approximately 3.5% of all benign primary bone tumors and 1% of all bone neoplasms. Osteoblastoma mostly affects the vertebral column and long bones and is rarely seen in the ribs (2, 3). The latest case of rib osteoblastoma has been described by Ye et al. (5). Frequently it lacks the characteristic pain of osteoid osteoma and could be misdiagnosed as other osteoblasic tumors by its rare location (3, 4). We present a case of rib osteoblastoma that was demonstrated by recurrence of the lesion.

## 2. Case Presentation

**Figure 1.**
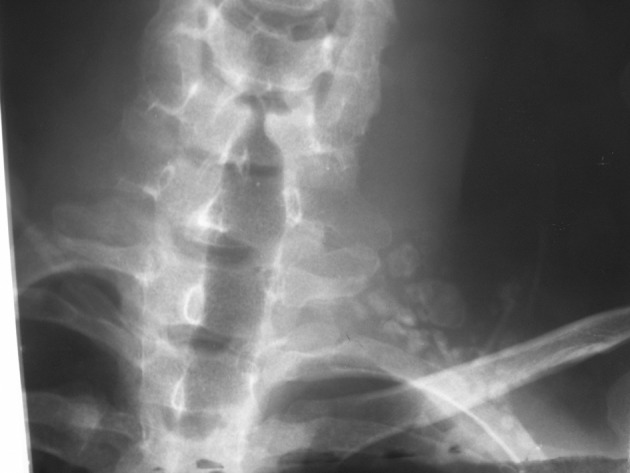
Chest X-ray of the patient shows a well-defined salient osseous expansion with spongy-form sclerotic areas on the posterolateral side of the first left rib.

**Figure 2.**
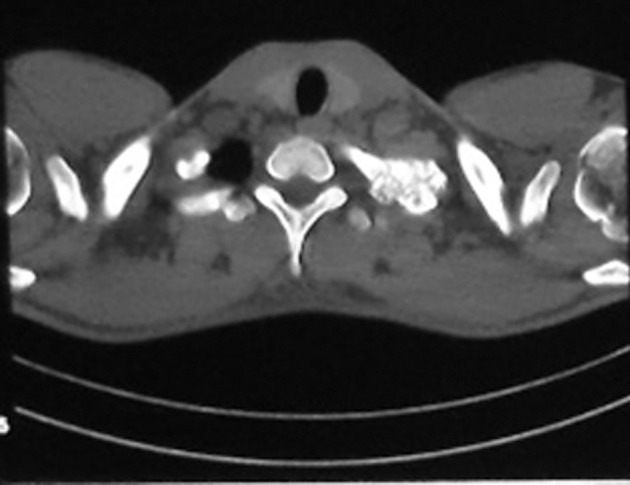
Non-contrast enhancement CT demonstrates an oval sclerotic, expansile lesion with spongy-form speckled calcification and a well-demarcated lesion involving the posterolateral side of the left first rib.

**Figure 3.**
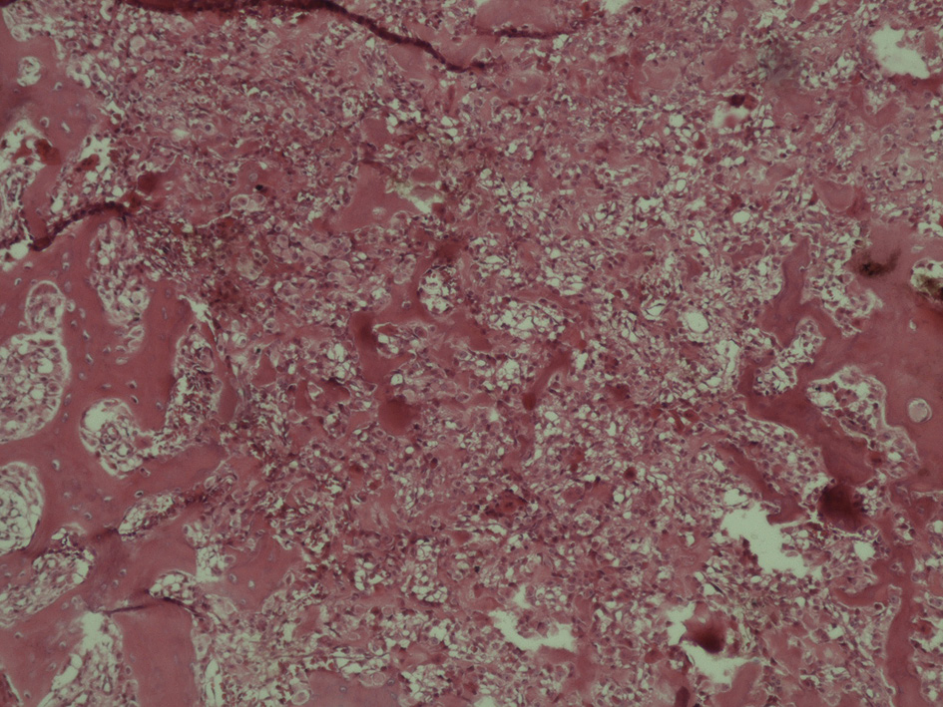
A bone trabeculae with a central nidus consisting of anastomosing bony trabeculae in a highly vascularized connective tissue (×20).

**Figure 4.**
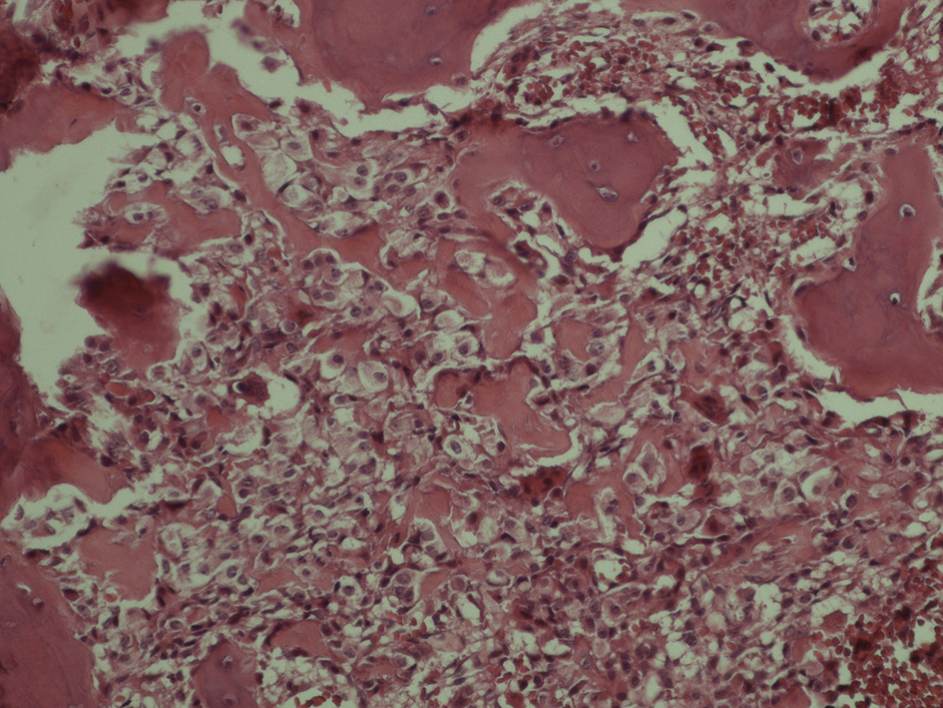
Variable osteoid calcifications lined by plump osteoblasts are also visible that are characteristic of osteoblastoma (×20).

**Figure 5.**
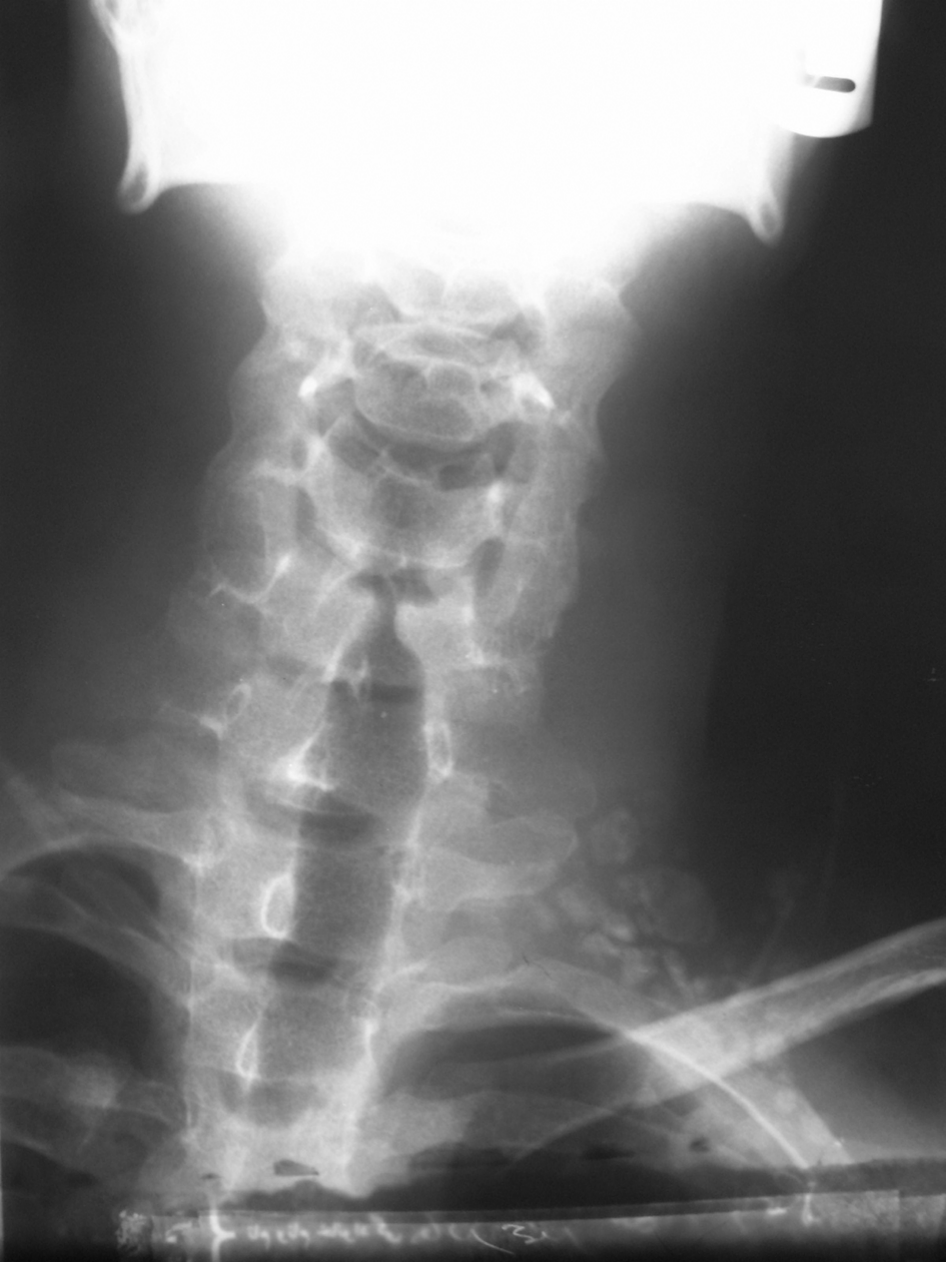
Neck and shoulder X-ray demonstrates geographic and well-circumscribed opacities.

**Figure 6.**
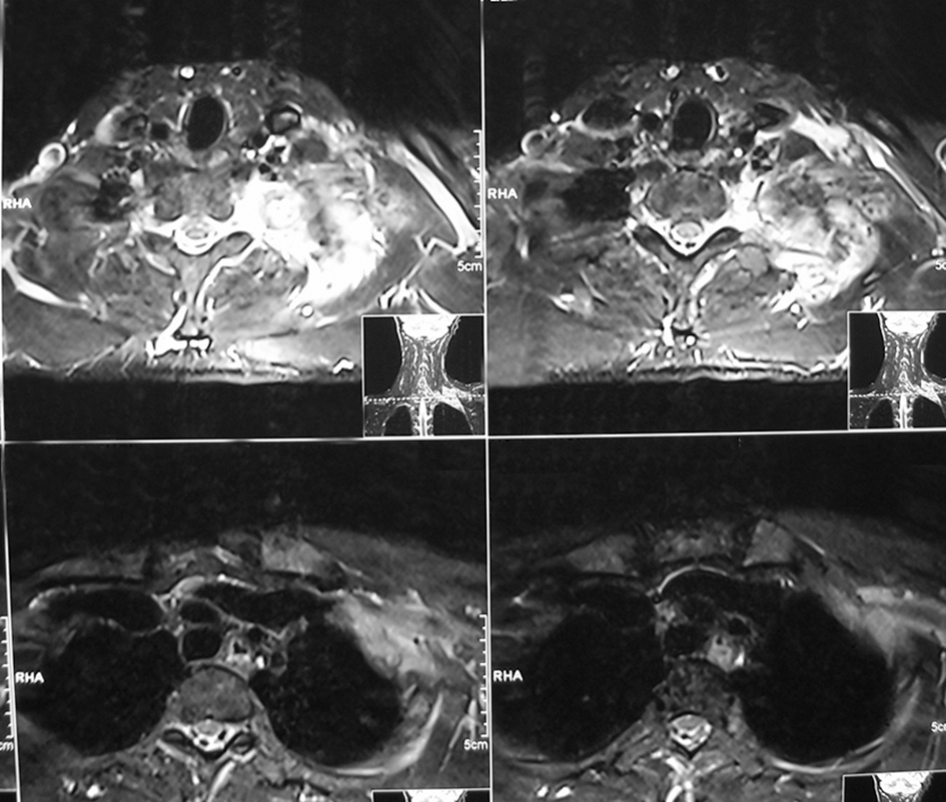
MRI reveals extensive edema around the lesion and high signal intensities on T2WI with punctate low signal areas consistent with mineralization on T1WI.

**Figure 7.**
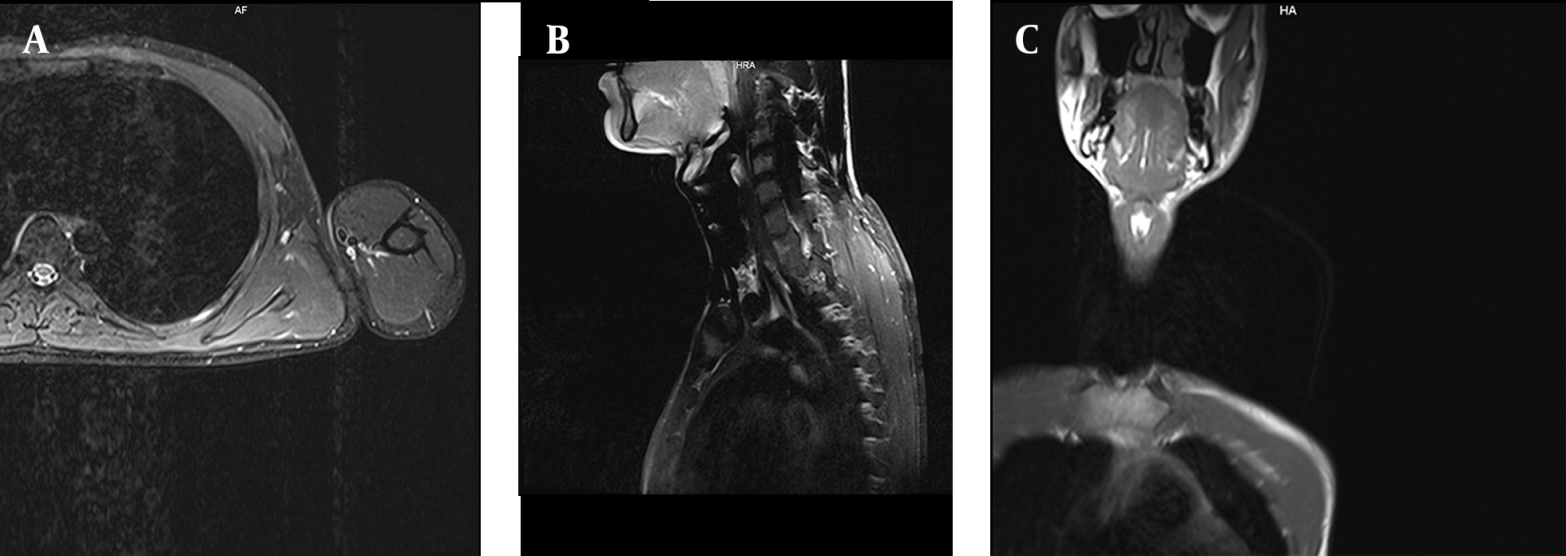
A, Axial fast spin-echo T2W image. B, Sagittal fast spin-echo T1W image. C, Post-gadolinium coronal T1W image

A 22-year-old man came with a long history of neck stiffness, back and neck pain radiating to his left upper extremity. On physical examination, no swelling was found, but there was a mild tenderness around the left side of his neck and shoulder. The symptoms had begun since 2 years ago and did not alleviate by pain killers such as salicylates. On a routine chest X-ray, the significant lesion involved the posterolateral aspect of the first left rib. Lordotic view chest X-ray demonstrated an expansile, oval, sclerotic mass with well-defined borders and speckled calcification without any internal lucency and periosteal reaction (Figure 1). In computed tomography (CT) scan the lesion appeared as an oval, well-demarcated tumor involving the left first rib. The sclerotic lesion was expanded by sharp margins, internal bone matrix and there was no evidence of periosteal reaction or a soft tissue mass. The size of the salient tumor was 3.59 × 2.60 cm (Figure 2). The patient underwent surgical resection to rule out osteosarcoma and alleviate his chronic progressive pain. Pathologically, the excised lesion composed of multiple pieces of brownish colored tissues with gritty, bony and friable consistency 8×3×2 cm in size. Histopathologic examination revealed bone trabeculae with a central nidus consisted of anastomosing bony trabeculae in a highly vascularized connective tissue (Figure 3). Exuberant new osteoid and bone trabeculae with variable calcification lined by plump osteoblasts were also visible that was characteristic of osteoblastoma (Figure 4). No other treatment was considered for the patient, but after six months he came with pain at the same area. Neck and shoulder X-ray demonstrated geographic well-circumscribed opacities adjacent to the primary lesion in the previous imaging, which was on the first left rib (Figure 5). The nature of the lesion conformed to ossification as well as mineralization. At that time, magnetic resonance (MR) imaging on T2WI showed high signal intensities around the lesion compatible with edema and punctate foci of signal void intensities consistent with mineralization on T1WI without any enhancement on post-contrast imaging (Figure 6). Three months later, another MRI was ordered. In the new MRI, lobulated mass-like lesions with low signal intensities on coronal T1WI and high signal intensities on axial T2WI, abutting upon the left apex of the hemithorax were demonstrated. On sagittal T1WI post-gadolinium contrast, it appeared as multiple small, round mass-like lesions with fine nodular and peripheral enhancement (Figure 7). The mentioned findings on MRI and X-ray could be related to the relapse of osteoblastoma due to an aggressive form or myositis ossificans. After wide resection, histopathological examination again revealed benign osteoblastoma. 

## 3. Discussion

Osteoblastoma is a rare osteoblastic benign bone tumor that most frequently affects the vertebral column and sacrum, but it may virtually affect any bone in the body (1, 2). Several studies demonstrate 34% long tubular bone involvement and 30% vertebral bone involvement. Additionally, the bones of the lower extremity are involved in 35% of cases, the bones of the upper extremity in 9% and the skull, mandible or maxilla in 15%. When located in the long tubular bones, 75% are situated in the diaphysis and the remainder in the metaphysis (6, 7). Involvement of other bones such as tarsal bones and clavicle have been reported and the rib involvement is very rare (7). Pain in osteoblastoma is often progressive, accompanied by other signs such as local swelling, tenderness and warmth that will not be relieved by salicylates such as aspirin that is characteristic of osteoid osteoma (7, 8). Our patient’s pain did not alleviate by salicylates as well. Males are affected more than females in a ratio of 2.5-1. The adjacent bone to the benign osteoblastoma is often not sclerosed (6-8). The radiographic features are varied and often non-diagnostic, but have characteristics similar to osteoid osteoma, depicting a normal or oval, well-circumscribed lytic defect that is surrounded by a zone of reactive sclerosis (9). In our case, the relevant lesion radiographically demonstrated features of osteoblastoma consisting of osseous expansion and well-defined spongy form sclerosis without any internal lucency. CT scan is often necessary to help clinical and routine radiographic findings that are suggestive of osteoblastoma to detect a nidus with matrix mineralization and also definitive detection of tumor margins for further surgery (9). MRI helps detect nonspecific reactive marrow and soft tissue edema, and exactly defines soft tissue extension (9). In our case, MRI was not done before surgery. In nearly 5% of all cases, radiographs show patchy or cloud-like radiopacities. It may expand the bone contour with a markedly thinned cortex or could be destroyed focally, with or without periosteal reaction (8, 9). Thus, these findings may produce confusion with malignant lesions in the radiological diagnosis that may occur in 20% of osteoblastomas. For these reasons, in the differential diagnosis, osteosarcoma could be considered. In our case, we could not find such equivocal findings in the initial studies. Bone scintigraphy demonstrates radionuclide accumulation at the site of the lesion. Histologically, osteoblastoma may have foci of neoplastic epithelial cells with bizarre nuclei admixed with telangiectatic appearance that resembles osteosarcoma and differentiation of these two entities is highly valuable for patient treatment. On the other hand, radiography helps the pathologist differentiate these conditions (10, 11). Osteosarcoma, osteoid osteoma, and aneurysmal bone cyst are the histopathological differential diagnoses of osteoblastoma (11). Ye et al. (5) reported osteoblastoma in the posterior shaft of the right fifth rib in a 59-year-old female with a history of right back pain for two years and the radiologic impression was osteochondroma (5). In our case, osteochondroma was one of the radiologic differential diagnoses, though the radiographic findings were not specific for osteoblastoma and may be misdiagnosed as other benign bone tumors. Wimpee et al. (12) reported an 8-year-old female with a previous history of scoliosis and after investigations they found stage III benign osteoblastoma of the rib as a predisposing factor (12). In the reported cases, the lesions demonstrated by chest X-ray with characteristic features for osteoblastoma were not seen. So the lesion were excised for precise histopathological diagnosis. In our case, the mentioned imaging findings were not specific, so surgeons operated on the patient for definite diagnosis. Intralesional curettage is the treatment of choice in benign osteoblastoma, followed by further wide resection to prevent the rate of recurrence that is not unusual in osteoblastoma (7). Our case came again with dull pain and a tumoral lesion in the same area. The lesion was excised because of suspicion of aggression. Presence of epithelioid osteoblastic cells with mitotic figures in the histopathological examination confirmed the benign nature of the lesion without an aggressive feature. Although both radiographic findings and recurrence were suggestive of an aggressive type tumor, our case suffered from a benign one. It is better to point out that the recurrent form of osteoblastoma is more common in the aggressive type (7, 10).
